# The Chemical Interplay between Nitric Oxide and Mitochondrial Cytochrome *c* Oxidase: Reactions, Effectors and Pathophysiology

**DOI:** 10.1155/2012/571067

**Published:** 2012-07-01

**Authors:** Paolo Sarti, Elena Forte, Alessandro Giuffrè, Daniela Mastronicola, Maria Chiara Magnifico, Marzia Arese

**Affiliations:** ^1^Department of Biochemical Sciences and Istituto Pasteur-Fondazione Cenci Bolognetti, Sapienza University of Rome, Piazzale Aldo Moro 5, 00185 Rome, Italy; ^2^CNR Institute of Molecular Biology and Pathology, Piazzale Aldo Moro 5, 00185 Rome, Italy

## Abstract

Nitric oxide (NO) reacts with Complex I and cytochrome *c* oxidase (CcOX, Complex IV), inducing detrimental or cytoprotective effects. Two alternative reaction pathways (PWs) have been described whereby NO reacts with CcOX, producing either a relatively labile nitrite-bound derivative (CcOX-NO_2_^−^, PW1) or a more stable nitrosyl-derivative (CcOX-NO, PW2). The two derivatives are both inhibited, displaying different persistency and O_2_ competitiveness. In the mitochondrion, during turnover with O_2_, one pathway prevails over the other one depending on NO, cytochrome *c* ^2+^ and O_2_ concentration. High cytochrome *c* ^2+^, and low O_2_ proved to be crucial in favoring CcOX nitrosylation, whereas under-*standard* cell-culture conditions formation of the nitrite derivative prevails. All together, these findings suggest that NO can modulate physiologically the mitochondrial respiratory/OXPHOS efficiency, eventually being converted to nitrite by CcOX, without cell detrimental effects. It is worthy to point out that nitrite, far from being a simple oxidation byproduct, represents a source of NO particularly important in view of the NO cell homeostasis, the NO production depends on the NO synthases whose activity is controlled by different stimuli/effectors; relevant to its bioavailability, NO is also produced by recycling cell/body nitrite. Bioenergetic parameters, such as mitochondrial ΔΨ, lactate, and ATP production, have been assayed in several cell lines, in the presence of endogenous or exogenous NO and the evidence collected suggests a crucial interplay between CcOX and NO with important energetic implications.

## 1. Introduction

It is nowadays established that nitrogen monoxide (NO), nitric oxide in the literature, inhibits mitochondrial respiration. The inhibition is induced by the reaction of NO with some of the complexes of the respiratory chain, according to mechanisms studied over more than 20 years. The reaction of NO with Complex III is sluggish [1], whereas the reaction of NO with Complex I and Complex IV, that is, cytochrome *c* oxidase (CcOX), is rapid and to a large extent reversible. Both reactions lead to formation of derivatives responsible of the mitochondrial nitrosative stress observed in different pathophysiological conditions, including main neurodegenerations [2–6]. The functional groups of the mitochondrial complexes reacting with NO include the metals at the catalytic active site of CcOX, namely, the Fe and Cu ions of the heme *a*_3_-Cu_B_ site [7, 8]. The inhibition of Complex I results from the reversible S-nitrosation of Cys39 exposed on the surface of the ND3 subunit [9, 10]. The functional effects on cell respiration depend on the complex targeted by NO and on type of reaction. Inhibition of both Complex I and CcOX is mostly reversible, becoming irreversible, however, depending on duration of the exposure to NO and on its concentration [10, 11]. The onset of NO inhibition on Complex I is slow (minutes [10]), whereas on CcOX is very fast (milliseconds to seconds [12]). In this paper the attention is focused on the interactions between NO and CcOX. The balance between the concentrations of cytochrome *c* ^2+^ and O_2_ proved to be critical in inducing different CcOX inhibition patterns, spanning from a finely tuned *control *to a severe, almost irreversible enzyme inactivation [13]. The interplay between CcOX and NO is based on the inhibition exerted by NO on the enzyme that, in turn, actively controls the NO concentration at the mitochondrial site [14].

The redox active site of CcOX contains one heme *a*_3_ and one Cu_B_ tightly coupled in the so-called binuclear site, where the O_2_ and NO chemistry as well as the reaction with common ligands occur. The active site receives electrons intra-molecularly from the reduced heme *a* and Cu_A_, forming together the electron accepting pole of CcOX, maintained physiologically reduced by cytochrome *c*. Also relevant to the reaction of NO with CcOX, the availability in the mitochondrion of reduced cytochrome *c* depends on the relative rate at which it is reduced by Complex III and oxidized by O_2_  via CcOX. It is also worth mentioning that the absolute cytochrome *c* concentration may vary in different cell lines and tissues [15]. The rate of reaction of CcOX with O_2_ is close to diffusion limited (*k* ≈ 1 × 10^8^ M^−1^ s^−1^ [16, 17]), whereas the reaction with cytochrome *c* is slower, *k* ≈ 1 × 10^6^ M^−1^ s^−1^, the actual rate constant value being dependent on pH and ionic strength [18]. During turnover, the reduction level of the CcOX redox sites, and particularly of the metals in the active site, depends on (i) the actual concentration of reduced cytochrome *c* and O_2_ (weighted for their relative *K_M_* values) at the redox competent sites and (ii) the internal electron transfer rate from the electron accepting pole (heme *a*-Cu_A_), where cytochrome *c* reacts, to the active (heme *a*_3_-Cu_B_) site, where the O_2_ reaction takes place. At saturating concentration of the physiological substrates, the rate limiting step in the CcOX catalytic cycle is the internal electron transfer [19–21]. Over and above the description of the reaction mechanisms, the aim of this work is to stress the idea that CcOX uses both O_2_ and NO as physiological substrates [5, 14, 22, 23] and to review the experimental evidence pointing to a central role of the NO interplay with CcOX in cell bioenergetics.

## 2. CcOX Binds Reversibly or Oxidizes NO to Nitrite at the Active Site Where O_2_ Binds

In order to better understand the reciprocal interactions between CcOX and NO, it may help summarizing the intermediates populated by CcOX during turnover with physiological substrates. During the catalytic cycle the fully oxidized (**O**) heme *a*_3_-Cu_B_ site accepts a first electron from Cu_A_/heme *a*, leading to formation of a partially, single-electron, reduced (**E**) species; subsequently, a second electron is transferred to the active site, and the fully reduced (**R**) species is formed. Once in the **R** state, O_2_ binds rapidly generating the short-lived (microseconds, at 20°C) compound **A**, in which O_2_ is complexed to heme *a*_3_^2+^ [24]. Electrons are rapidly delivered to bound O_2_, and Compound **A** converts to a nominal peroxy (**P**) complex with both heme *a*_3_ and Cu_B_ oxidized; actually, the experimental evidence suggests that the O–O (peroxy) bond in this **P** species is already cleaved off, showing heme *a*_3_ in the ferryl (Fe^4+^=O) form and a tyrosine residue in a radical state [25, 26]. By accepting a third electron, **P** decays quickly into a canonical ferryl (**F)** intermediate [27], that eventually converts back to the fully oxidized **O** state upon arrival of one last electron from Cu_A_/heme *a*. The sequential steps and the intermediates populated during a single turnover are indicated, starting and ending with the fully oxidized **O **species:


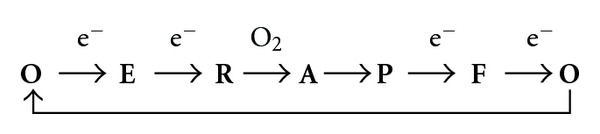


Since first proposed as a unified picture based on experiments carried out using purified CcOX [28], the enzyme adducts formed upon reacting with NO have been spectroscopically identified as a nitrosyl-derivative (heme *a*_3_^2+^-NO) or as a nitrite-bound (heme *a*_3_^3+^-NO_2_^−^) derivative, or a mixture of these two species, depending on the steady-state fractional accumulation of all the intermediates [29]. It is worthy to notice that the Fe and Cu ions in the active site undergo redox changes only upon reacting in the oxidized state (i.e., Fe^3+^, Fe^4+^, Cu^2+^) with NO. During the reaction NO is oxidized to NO_2_^−^, that is released in the medium; the whole event is identified as pathway 1 (PW1):


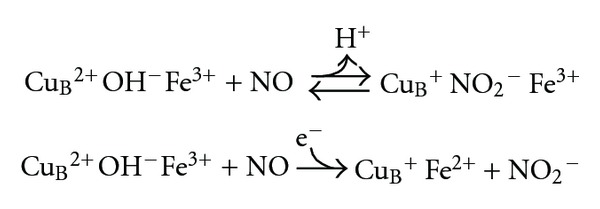


Otherwise, if the active site is partially or fully reduced, an affinity-driven NO binding to these metals takes place; the whole event is identified as pathway 2 (PW2) and occurs without further redox events:


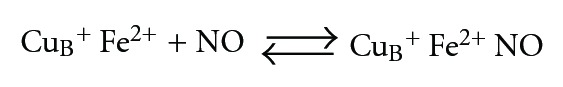


**Figure 1:**
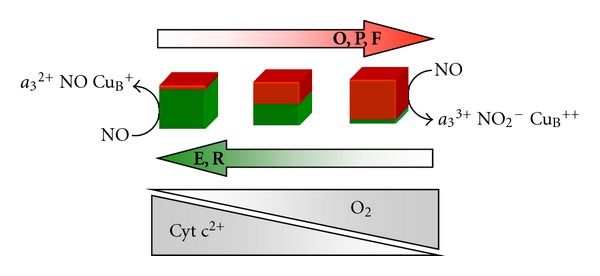
Dual-pathway model for the interaction of NO with mitochondrial cytochrome oxidase. The nature of the interaction between NO and CcOX depends on the catalytic intermediates targeted, and these are differently populated at different concentrations of O_2_ and reduced cytochrome *c*. The oxidized intermediates **O**, **P**, **F** (see text) are overall more populated with increasing O_2_ availability, and/or decreasing the concentration of reduced cytochrome *c* in the mitochondrion: these intermediates react with NO generating a nitrite-inhibited CcOX. The reduced species **E** and **R** (see text) buildup, instead, upon decreasing O_2_ and/or increasing the concentration of reduced cytochrome *c*: upon reacting with NO, these intermediates generate a heme *a*_3_^2+^-NO complex, in competition with oxygen.

NO is very reactive towards the fully reduced **R** binuclear site. It binds to heme *a*_3_^2+^ at a rate similar to that of O_2_, that is, *k* = 0.4 − 1 × 10^8^ M^−1^ s^−1^ [16, 17], yielding the high affinity Fe^2+^ nitrosyl adduct, whose accumulation is observable directly by spectroscopy or indirectly by NO amperometry [30, 31], when the fully reduced CcOX in detergent solution is mixed with NO. Interestingly, in the presence of NO, all circumstances favoring the electron donation to the catalytic site of CcOX or slowing down its oxidation by O_2_ as during hypoxia (i.e., when the [O_2_] ≤  K_M,O_2__  of CcOX ) proved to favor CcOX nitrosylation [32].  Figure 1 shows schematically how accumulation of the turnover intermediates correlates with the build up of the nitrosylated (Cu_B_^+^ Fe^2+^NO) or the nitrite-bound (Cu_B_^+^NO_2_^−^ Fe^3+^) species.

It is worth mentioning that, contrary to a few bacterial oxidases [34–36], mitochondrial CcOX cannot reduce to N_2_O the NO bound at reduced heme *a*_3_ [30]. This implies that the functional recovery of the enzyme after NO binding necessarily lags behind the thermal dissociation of NO from the active site. The dissociation reaction is relatively slow (*k*_off_ = 3.9 × 10^−3^ s^−1^ at 20°C) and photosensitive [28]. Photosensitivity has been widely used by Sarti and coworkers to gain insight, through amperometric measurements, into the mechanism of CcOX inhibition by NO in mitochondria or whole cells [37], that is, under conditions unfavorable to spectroscopy. Since the fully reduced binuclear site reacts eagerly with both O_2_ and NO, the inhibition of CcOX via formation of a nitrosyl adduct is expected to occur in competition with O_2_, that is, according to PW2. Consistently, the O_2_ competition is more clearly observed when the concentration of the reducing substrates favors the reduction of the enzyme [29, 32]. In any case, the inhibition of the nitrosylated CcOX is reverted at the rate of the NO thermal dissociation from reduced heme *a*_3_ [28]. It is worth noticing that, although the NO dissociation process is mechanistically independent of O_2_ concentration, bulk O_2_ shortens the duration of inhibition by oxidizing free NO in solution, thus hampering NO rebinding to CcOX.

## 3. The Fully- and Half-Reduced Binuclear Site

The ability of the single electron reduced **E** species to bind NO was investigated using the K354M mutant of the *Paracoccus denitrificans* CcOX [38]. In this mutant the internal electron transfer from the electron accepting pole to the active site is severely impaired, so that the full reduction of the active site and its reaction with O_2_ is achieved very slowly, that is, within several minutes. Under these conditions the electron transferred intramolecularly from heme *a*/Cu_A_ resides on either heme *a*_3_ or Cu_B_, and the resulting **E** species can not react with O_2_. Interestingly, however, **E** reacts promptly with NO generating the nitrosyl derivative. Thus, one can conclude that, unlike O_2_, NO binds to the binuclear active site even before its complete reduction [12, 31]. Whether the reaction with **E** plays a role in the mechanism of CcOX inhibition by NO during turnover is still unclear, since it has been also suggested that at steady-state the reaction of NO with **E** is not required to account for fast inhibition [32, 39]. Regardless of whether the reaction of NO with either **E** or **R** is predominant, it seems feasible to conclude that all conditions leading to reduction of the binuclear site in the presence of NO favor nitrosylation of the enzyme.

## 4. The Role of Cu_**B**_ in the Reaction with NO

The reaction of NO with Cu_B_ in the fully oxidized CcOX to form nitrite was first reported by Brudvig and coworkers in the early 80s [40]. Later on this reaction was reinvestigated by Cooper et al. [41] and Giuffrè et al. [42], using a *pulsed* (*fast*) preparation of CcOX. The pulsing procedure that *in vitro* consists in preliminary reduction-reoxidation of CcOX [43], removes chloride from the oxidized active site of the enzyme thereby allowing fast reaction with NO [42]; indeed, CcOX is expectedly in the pulsed state *in vivo* where CcOX turnover takes place continuously. During the reaction with the oxidized Cu_B_ (*k* = 2 × 10^5^ M^−1^ s^−1^ at 20°C), NO is transiently oxidized to nitrosonium ion (NO^+^), which is subsequently hydroxylated (or hydrated) to nitrite/nitrous acid.

Thus, after the reaction, the enzyme displays nitrite bound to ferric heme *a*_3_ and is inhibited. The affinity of nitrite for the reduced heme *a*_3_, however, is much lower than the affinity for the oxidized active site. The intramolecular electron transfer to heme *a*_3_-Cu_B_, therefore, causes the prompt dissociation of nitrite and the subsequent full restoration of activity [29, 44]. Relevant to possible pathophysiological effects of CcOX inhibition by NO, it is worthy to notice that the nitrite dissociation upon reduction of heme *a*_3_ (*k*~6 × 10^−2^ s^−1^ at pH = 7.3, *T* = 20°C [29]) is approximately one order of magnitude faster than the NO-dissociation from the nitrosylated site, accounting also for the observed production of nitrite by isolated mitochondria [45, 46].

It has been proposed that nitrite formation could follow an alternative route via reaction with O_2_ of the NO bound to the fully reduced CcOX [46]. According to this proposal, a superoxide anion (O_2_^−^) forms by the reaction of O_2_ with reduced Cu_B_ and reacts with NO bound to reduced heme *a*_3_ to yield peroxynitrite; peroxynitrite is reduced in turn by the enzyme to nitrite, which is finally released in the bulk. The hypothesis, though feasible and intriguing, was not confirmed by independent experiments specifically designed to investigate the kinetics and the products of the reaction of fully reduced nitrosylated CcOX with O_2_ [50]. Using myoglobin as an optical probe for free NO, the NO bound to reduced heme *a*_3_ was shown to be displaced by excess O_2_ at the low rate of thermal dissociation, to be eventually released in the bulk as such, and not as nitrite [50]. The NO dissociation from the heme iron takes minutes, also when assayed in mitochondria or intact cells, at 37°C and in the dark, that is, under conditions common *in vivo* in internal organs and tissues. The slow recovery of function of the nitrosylated CcOX is compatible with a more severe state of inhibition characteristic of PW2.

The role of Cu_B_ in the CcOX-mediated oxidation of NO to nitrite was also addressed in experiments carried out using the *E. coli* cytochrome *bd*. This oxidase lacks Cu_B_ and, consistently, reacts with NO much more slowly (*k* = 1.5 × 10^2^ M^−1^ s^−1^ at 20°C) than mitochondrial CcOX, without forming nitrite [51]. Interestingly, the NO dissociation from the Cu_B_-lacking cytochrome *bd* oxidase (from *E. coli*) is much faster [52, 53], pointing to a specific property of heme *d* [54] and/or to a role of Cu_B_ also in the NO dissociation from the active site. As a matter of fact, this peculiarity was suggested to confer to cytochrome *bd*-expressing bacteria a higher resistance to nitrosative stress [53, 55, 56], a hypothesis supported by *in vitro* studies on *E. coli* deletion mutants of each of the two alternative respiratory oxidases (cytochrome *bd* and cytochrome *bo*_3_) [55].

## 5. Cells Respiring in the Presence of NO and Using Endogenous Substrates

The respiration of cells grown under *standard* conditions, that is, in the presence of (unlimited) O_2_ and endogenous reducing substrates, is inhibited by NO but without detectable accumulation of nitrosylated CcOX [37, 57]. As a matter of fact, these standard culture conditions favor the overall accumulation of the CcOX intermediates **P**, **F **and **O** [29, 41, 42, 58]; these are the species responsible for the NO oxidation to nitrite. Consistently, upon rapid and efficient scavenging of bulk NO, respiration is promptly recovered. It is worthy to point out that nitrite, far from being a simple oxidation byproduct, represents a source of NO particularly important in view of the NO cell homeostasis [59–62]. When the oxygen tension decreases in tissues, not only respiration but also the production of NO by nitric oxide synthases (NOSs) is severely impaired, as the NOS uses O_2_ as cosubstrate [63]. Anoxia, however, induces tissue acidification, which promotes the reduction of nitrite to NO, compensating for impairment of the NOS-dependent NO production [59, 60, 64]. Consistently, and apparently important for a cardiovascular response, low doses of nitrite (~50 nM) administered to ischemic, heart-arrested mice, early during resuscitation procedures, were shown to significantly improve survival of the treated animals compared to controls [61].

The CcOX NO-inhibition pathway prevailing in mitochondria under given metabolic conditions might be responsible for pathological responses of cells and tissues [57]. Compelling experimental evidence has been collected suggesting that the O_2_-uncompetitive nitrite inhibition pathway (PW1) prevails under conditions of low electron flux through the respiratory chain and high O_2_, whereas the O_2_-competitive nitrosyl pathway (PW2) takes over as the electron flux increases and O_2_ concentration decreases [32, 37].

The main features of the two pathways can be summarized as follows:

- both reactions lead to the rapid accumulation of a CcOX inhibited species, characterized by different stability, *K*_*I*_, and O_2_ competitiveness (Table 1);
- one pathway prevails over the other one depending on the fractional accumulation of the NO-targeted CcOX turnover intermediates [28, 29], whose distribution depends in turn on the *in situ* availability of O_2_ and reduced cytochrome *c*; the concentration of the latter ultimately depends on its absolute concentration and on the electron flow level trough the respiratory chain;
- PW1 prevails under basal mitochondrial metabolic conditions;
- PW2 prevails under conditions favoring the accumulation of **E** and **R**, that is, when the concentration of cytochrome *c* ^2+^ at the CcOX site increases and/or the O_2_ tension decreases;
- the accumulation of CcOX-NO or CcOX-NO_2_^−^ affects differently the mitochondrial bioavailability of NO: the nitrosyl-derivative releases NO in the medium as such, that is, still reactive, whereas the nitrite-derivative releases nitrite to be further oxidized to nitrate, eliminated or rereduced to NO.

The NO concentration level in the cell varies depending on the relative rate of its production, and degradation or scavenging. Unless exogenously supplemented to the cells (NO-donors), the enzymatic endogenous NO production is controlled via the activation/inhibition of the cell NO-synthases. Alternatively, as mentioned above, NO is generated by the protein-bound or free metal ions (Fe^2+^, Cu^+^) catalyzed reduction of NO_2_^−^, a reaction that commonly occurs in solution, at acidic pH [59, 60]. The NO bioavailability can be lowered, therefore, by specific cell-permeable NO-synthase inhibitors or by NO scavengers, such as heme-proteins or reduced glutathione [65].

As pointed out by Cooper and Giulivi [5], when the NOS activity is inhibited, one may expect the O_2_ consumption by respiring mitochondria to increase. This event, however, has been often but not always observed [5], probably owing to the activation of alternative NO-releasing systems, such as nitrosoglutathione and S-nitrosated protein thiols, or the NO_2_^−^ reduction, all active regardless of the presence of NOS inhibitors.

## 6. Effectors and Pathophysiology

**Figure 2:**
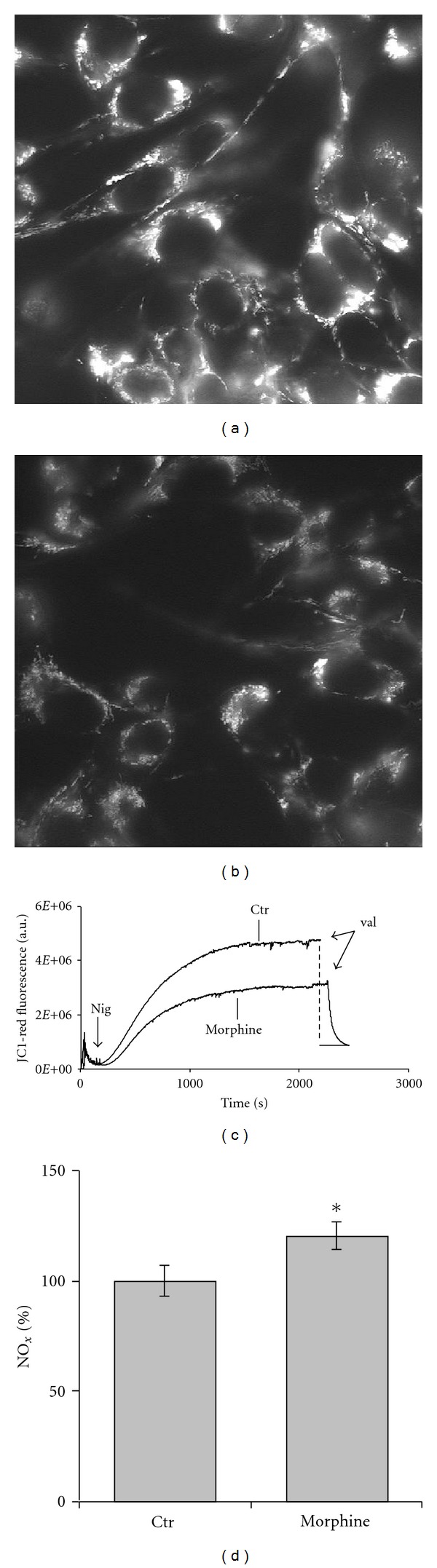
Morphine-induced mitochondrial membrane potential and NO*_x_* changes in Glioma cells. *Fluorescence microscopy*: control cells (a), 20 nM morphine incubated for 24 h (b). Mitochondrial membrane potential (ΔΨ) was probed using Rhodamine123; the dye is electrophoretically accumulated by the cell mitochondria. *Bulk fluorescence* (c). Control (ctr) versus morphine-treated (morphine) cells, assayed in air-equilibrated medium and in the presence of 2 *μ*M ouabain and 0.4 *μ*M JC-1; after signal stabilization, 0.6 *μ*M nigericin is added and fluorescence changes followed over time. Addition of valinomycin abolishes the membrane potential. Excitation and emission wavelength, 575 nm and 590 nm, respectively. *Nitrite accumulation* (d). The release of NO*_x_* (nitrite and nitrate) in the medium and during incubation with morphine was assessed spectrophotometrically by the Griess reaction; results expressed as percentage of control cells (*c*tr). *P < 0,05. Modified from [33].

**Figure 3:**
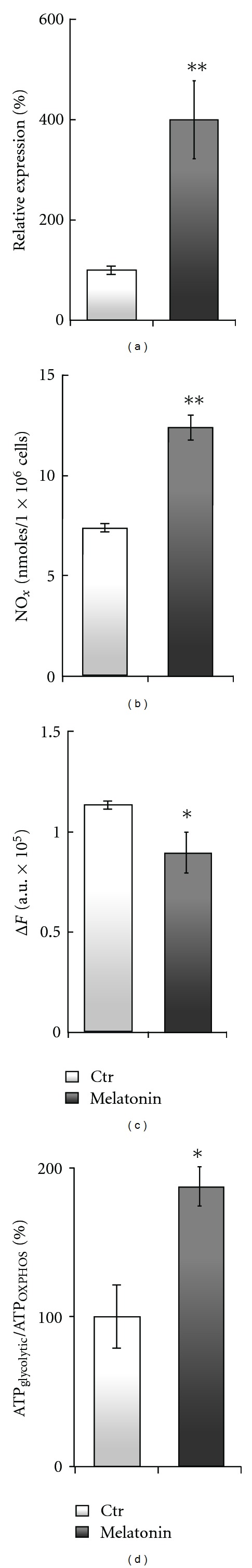
Melatonin-induced changes of the nNOS mRNA expression in HaCaT cells: effect on NO*_x_* production and mitochondrial membrane potential. (a)—Real-time PCR quantification of nNOS mRNA (*β*-actin gene used for normalization). (b)—Fluorometric determination of the NO*_x_* release in the cell culture medium. (c)—Mitochondrial membrane potential evaluated as the fluorescence difference, ΔF, from the maximal (*plateau*) to the minimal level reached after addition of valinomycin (see also Figure 2(c)). (d)—Contribution of oxidative phosphorylation and glycolysis to ATP production, directly evaluated according to [47]. The ATP_glycolytic_/ATP_OXPHOS_ ratio is indicative of the ability of a given cell line to compensate with glycolysis an OXPHOS impairment (so-called, Warburg effect) [48, 49]. The release of NO induced by melatonin almost doubles the glycolytic contribution to ATP synthesis in HaCaT cells. Cells were incubated with 1 nM melatonin for 6 h. (a), (b): **P < 0,01 versus CTR; (c), (d): *P < 0,05 versus CTR. Modified from [48].

Over the years, the enzymatic NO release has been induced in cultured cells, tissues, and organs, either using effectors able to activate cell Ca^2+^ fluxes [66], thus stimulating the constitutive NOS, or by enhancing the expression of the inducible isoform of NOS (iNOS) [67]. Morphine is the prototype of a family of drugs used in analgesia and cancer pain treatment [68, 69]. Relevant to the NO chemistry, morphine activates the opioid and the N-methyl-D-aspartate receptors of neuronal cells, triggering Ca^2+^ fluxes and NO release [70, 71]. In 2004, Mastronicola et al. [33] confirmed that the persistence of nanomolar morphine in the cell culture of glioma cells was able to induce the accumulation of nitrite/nitrate in the medium. Interestingly, the cell mitochondria displayed a membrane potential drop off, as probed by a significant decrease of the intramitochondrial JC-1 red-aggregates, whose accumulation requires high mitochondrial ΔΨ values (Figure 2) [72]. Thus, over the same time scale of a cell Ca^2+^ transient (seconds to minutes) the NOS activation can affect the mitochondrial potential [33]. More recently, Arese et al. [48] have shown a transient inhibition of the mitochondrial respiratory chain in human adult low calcium temperature (HaCaT) cells, maintained in a standard culture medium, in the presence of nanomolar (or less) melatonin. After a few hours incubation compatible with a receptor-mediated process [73], and with a timecourse compatible with the circadian melatonin biorhythm, the basal mRNA expression level of the neuronal NOS (nNOS) in the cells was raised by a factor of ~4 (Figure 3(a)), returning, thereafter, to basal level [48]. As shown in the same figure, within the same time scale, the authors observed that: (i) the production of nitrite and nitrate (NO*_x_*) was increased (Figure 3(b)) and (ii) the mitochondrial membrane potential was decreased (Figure 3(c)). Consistently, the ATP_ OXPHOS_ production was also decreased and an increase of glycolytic ATP and lactate was detected [48]. Taken together, all these findings suggest that mediated by the melatonin receptors, NO is released and CcOX is reversibly inhibited, with significant bioenergetic consequences. Since cells are not likely facing conditions compatible with the accumulation of CcOX intermediates **E** or **R**, we can infer that inhibition has occurred via PW1. Interestingly, therefore, under physiological conditions, within the limits of a cell culture, a few hours exposure to hormonal-like concentrations of melatonin is able to exert some inhibition on mitochondrial OXPHOS and to raise the ATP_glycolytic_/ATP_ OXPHOS_ ratio by a factor of ~2 (Figure 3(d)) as expected on the basis of a compensatory physiological Warburg effect [74]. All together these findings suggest that physiological concentrations of melatonin may play a mitochondrial role and interestingly in a circadian context. Indeed, the hypothesis that the melatonin-driven shift towards glycolysis might have a physiological role in the chemistry of the night rest, though attractive, is presently fully speculative, and remains to be investigated.

Based on the effects of melatonin and on the information collected about the NO inhibition of purified CcOX or mitochondria [75, 76], it is also tempting to speculate on how the mitochondrial state can affect the response to NO, particularly under conditions compatible with a limited, and transient raise of NO concentration. It is worthy to consider that isolated state 3 mitochondria proved to be inhibited by NO more effectively than state 4 mitochondria [75, 76]. This suggests that the sensitivity to NO inhibition increases with the electron flux level of the respiratory chain, and particularly with the turnover rate of CcOX; under these conditions the CcOX inhibition is oxygen competitive [32]. In state 3 mitochondria, therefore, and in the presence of suitable amounts of reduced cytochrome *c*, the fractional accumulation of the reduced (**E** and **R)** CcOX species is expected to increase; these species are promptly nitrosylated in the presence of NO. At low turnover rate, as in state 4, the oxidized catalytic intermediates (**O**, **P** and **F**) are expected to be more populated [29], and the NO inhibition predominantly occurs following PW1. Both in state 3 and state 4, if the NO concentration is low (e.g., subnanomolar), the fraction of CcOX inhibited is limited, and the depression of respiration is almost insignificant [77, 78], a finding consistent with an excess capacity of CcOX [79, 80]. When NO persists in the cell environment, as during a prolonged incubation with even low (nM) concentration of NO, and particularly if the turnover rate of CcOX is increased, a substantial inhibition of the respiratory chain is predictable and synthesis of ATP_OXPHOS_ decreases [81]. Under these conditions, glycolysis likely takes place to compensate for ATP loss [82].

## 7. How Does the NO/CcOX Interplay Turn into Pathology

As just mentioned, the transient inhibition of mitochondrial OXPHOS may induce a physiological, compensatory activation of glycolysis [74]. This original observation by Warburg was recently reproposed by Almeida et al. [83], to rationalize the energetic changes of astrocytes and neurons inhibited by NO. In this respect, it is worth considering that neurons, astrocytes, lymphoid, keratinocytes cells, and in general different cell lines may possess a different glycolytic compensatory capacity of coping with OXPHOS NO-inhibition [48, 57, 83]. All the evidence so far collected shows that under standard cell culture conditions, a pulse of NO leads to the accumulation of the CcOX-NO_2_^−^ derivative [37], which is able to immediately and fully recover its function, provided that free NO is scavenged in the mitochondrial environment. On the contrary, when CcOX nitrosylation is induced by (artificially) rising the electron flux level at the CcOX site or by allowing the cells to respire towards hypoxia ([O_2_] ≤ *K*_M,O2_), the respiratory chain remains inhibited for longer times at the CcOX site [28, 29, 32, 84]. It is worth recalling that indeed everything else being equal, the functional recovery of CcOX-NO is approximately 10–20 times slower than recovery of CcOX-NO_2_^−^. Thus, at least in a first approximation, it is feasible to propose that, compared to conditions promoting the formation of the CcOX-nitrite adduct, conditions favoring CcOX nitrosylation are expectedly more dangerous for cells, since causing a 10–20 times longer inhibition of the mitochondrial respiratory chain. One may indeed speculate that the compensatory glycolytic ATP synthesis might become insufficient, when CcOX is maintained nitrosylated for longer times.

In 2008 Masci et al. [57] characterized the mitochondria NO inhibition pattern of cells collected from patients affected by Ataxia Telangiectasia (AT). This is a multisystemic genetic human disorder characterized by a conjunctival telangiectasia and by a cerebellar degeneration leading to progressive ataxia [85, 86]. The disease is caused by mutations of the AT-mutated gene (ATM), coding for a nuclear 350 kDa protein that controls cell cycle and DNA damage repair [87–89]. AT patients are characterised by a genetic instability and vulnerability to radiation-induced oxidative stress [90–94]. Compared to control cells, AT cells display a defective reactive oxygen species (ROS) scavenging capacity [95, 96], with a decreased bioavailability of reduced glutathione [96].

**Figure 4:**
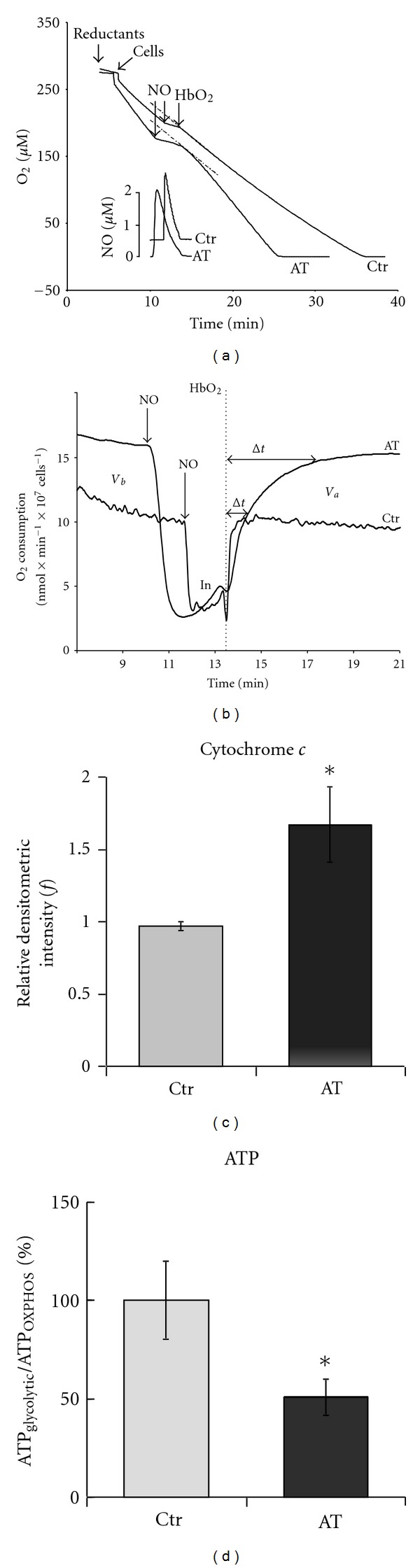
Oxygen consumption of Ataxia Telangiectasia (AT) cells: the inhibitory effect of NO. (a)—O_2 _consumption profiles of AT and control lymphoblastoid cells, recorded in the dark and in the presence of excess ascorbate and tetramethyl-p-phenylenediamine (TMPD). Inhibition of respiration was induced by adding a single bolus of pure NO gas solution (see lower NO profiles). In order to assess the fraction of residual inhibited CcOX-NO, the instantaneous rate was measured at 45 s after HbO_2_ addition. (b)—First derivative plots (integration time *t* = 2 s). Rate of O_2_ consumption before addition of NO (*V*_*b*_), and after addition of oxygenated hemoglobin, HbO_2_ (*V*_*a*_), that is, in the absence of free NO. *In*: inhibited state (in the presence of free NO). The Δ*t*  value is the time necessary for complete recovery of activity after addition of HbO_2_. *T* = 25°C. (c)—Cytochrome *c *immunoblot. Cell-lysate (30 **μ**g/well) of AT patients and controls (ctr). (d)—Relative contribution of OXPHOS and glycolysis to ATP production in AT and control cells. Modified from [57].

Relevant to a possible pathological implication of the NO mitochondrial inhibition, AT patients show a bioenergetic deficiency [97]. The mitochondrial functional characterization, and the NO inhibition pattern of lymphoid cells collected from AT patients, proved to be significantly altered. Based on the rate of respiration recovery from inhibition, under otherwise identical conditions of substrates availability (O_2_ and reductants), the CcOX in AT cells underwent nitrosylation to a substantially higher extent than in control cells [57]. As expected, based on the higher stability of the nitrosyl-adduct compared to the nitrite-adduct, after NO inhibition and subsequent removal of free NO, recovery of respiration of AT cells is slow, occurring at the rate of the NO displacement from the reduced CcOX active site, whereas control cells recover almost immediately (Figures 4(a) and 4(b)). As a matter of fact the inhibition of AT cells respiration was promptly removed upon shedding light on the cells (photosensitivity of the nitrosyl-adduct!). This peculiarity of AT cells has been correlated to their 1.7 fold higher concentration of mitochondrial cytochrome *c* compared to control cells (Figure 4(c)) [57]. The whole picture is consistent with the hypothesis that in AT cells, showing a lower ATP_glycolytic_/ATP_OXPHOS_ ratio compared to control cells (Figure 4(d)), the formation of **E** and **R** and thus CcOX nitrosylation is favored owing to the higher availability of reduced cytochrome *c* [29, 32].

## 8. The Dark Side of the Interplay between NO and CcOX

 In conclusion, regardless of the pathway leading to inhibition of CcOX, in the presence of NO, mitochondrial OXPHOS is impaired to some extent. Impairment is due to the slow displacement of NO from the active site or to the involvement of the site in the NO oxidation to nitrite. The evidence so far collected suggests that, if NO remains available in the mitochondrial environment, the mitochondrial membrane potential decreases, and glycolysis begins to contribute significantly to ATP synthesis. Thus, it seems crucial that cells responding to NO pulses are endowed with an efficient glycolytic machinery able to compensate for the decreased aerobic ATP production [82, 83].

Finally, let us consider for the sake of the argument a chronic hypoxia induced by an impaired microcirculation, for instance in the brain. Under these conditions common to many age-related neurodegenerations, one might expect an increased NO release to enhance the blood flow in response to hypoxia. In this already pathological scenario, however, the blood flow and thus O_2_ concentration may not increase significantly, owing to the vessel sclerosis; neurons could rather become hypoxic and in the presence of an increased NO concentration. These are the circumstances favouring PW2 (CcOX nitrosylation), even more so if the respiratory chain concentration of reducing substrates is still large enough. Under these conditions and in the absence of a suitable glycolytic compensation, the ATP levels could decrease dramatically, leading to cell death.

## Figures and Tables

**Table 1:** Cytochrome *c* oxidase versus NO—kinetic and thermodynamic parameters.

CcOX intermediate	CcOX adduct formed	*K*_*I*_ (nM)	*k*_ on_ (M^−1^ s^−1^) (*T* = 20°C)	*k*_ off_ (s^−1^) (*T* = 20°C)	O_2_-competition
**E**, **R**	Nitrosylated CcOX-NO	0.2 [32]	0.4–1 × 10^8^ [16, 17]	3.9 × 10^−3^ [28]	yes
**O,** **P**, **F**	Nitrite bound CcOX-NO_2_^−^	20 [32]	~1 × 10^5^ (**O**, **P**) ~1 × 10^4^ (**F**) [29, 58]	6.0 × 10^−2^ [29]	no

## Abbreviations

CcOX: Cytochrome *c* oxidase
CcOX-NO: Nitrosyl cytochrome *c* oxidase derivative
CcOX-NO_2_^−^: Nitrite-bound cytochrome *c* oxidase
PW1: NO reaction pathway leading to nitrite-bound CcOX
PW2: NO reaction pathway leading to nitrosyl CcOX
OXPHOS: Oxidative phosphorylation
ΔΨ: Membrane electrical potential difference
**O**: Fully oxidized CcOX
**E**: CcOX with single-electron reduced heme *a*_3_-Cu_B_
**R**: CcOX with fully reduced heme *a*_3_-Cu_B_
**A**: CcOX with ferrous oxygenated heme *a*_3_
**P**: ‘‘Peroxy” CcOX intermediate
**F**: ‘‘Ferryl” CcOX intermediate
NOS: Nitric oxide synthase
nNOS: Neuronal NOS
NO*_x_*: Nitrite-nitrate
AT: Ataxia Telangiectasia
HaCaT: Human adult low calcium temperature, that is, keratinocytes cell line
HbO_2_: Oxygenated haemoglobin
State 3 respiration: Induced by ADP, causing a burst of O_2_ consumption and ATP synthesis and relaxing into the slower State 4 respiration after ADP consumption.

## References

[1] Poderoso JJ, Carreras MC, Lisdero C, Riobó N, Schöpfer F, Boveris A (1996). Nitric oxide inhibits electron transfer and increases superoxide radical production in rat heart mitochondria and submitochondrial particles. *Archives of Biochemistry and Biophysics*.

[2] Moncada S, Erusalimsky JD (2002). Does nitric oxide modulate mitochondrial energy generation and apoptosis?. *Nature Reviews Molecular Cell Biology*.

[3] Blandini F, Braunewell KH, Manahan-Vaughan D, Orzi F, Sarti P (2004). Neurodegeneration and energy metabolism: from chemistry to clinics. *Cell Death and Differentiation*.

[4] Shiva S, Oh JY, Landar AL (2005). Nitroxia: the pathological consequence of dysfunction in the nitric oxide-cytochrome c oxidase signaling pathway. *Free Radical Biology and Medicine*.

[5] Cooper CE, Giulivi C (2007). Nitric oxide regulation of mitochondrial oxygen consumption II: molecular mechanism and tissue physiology. *American Journal of Physiology*.

[6] Erusalimsky JD, Moncada S (2007). Nitric oxide and mitochondrial signaling: from physiology to pathophysiology. *Arteriosclerosis, Thrombosis, and Vascular Biology*.

[7] Sarti P, Giuffrè A, Barone MC, Forte E, Mastronicola D, Brunori M (2003). Nitric oxide and cytochrome oxidase: reaction mechanisms from the enzyme to the cell. *Free Radical Biology and Medicine*.

[8] Cooper CE, Brown GC (2008). The inhibition of mitochondrial cytochrome oxidase by the gases carbon monoxide, nitric oxide, hydrogen cyanide and hydrogen sulfide: chemical mechanism and physiological significance. *Journal of Bioenergetics and Biomembranes*.

[9] Galkin A, Moncada S (2007). S-nitrosation of mitochondrial complex I depends on its structural conformation. *Journal of Biological Chemistry*.

[10] Clementi E, Brown GC, Feelisch M, Moncada S (1998). Persistent inhibition of cell respiration by nitric oxide: crucial role of S-nitrosylation of mitochondrial complex I and protective action of glutathione. *Proceedings of the National Academy of Sciences of the United States of America*.

[11] Cooper CE, Davies NA, Psychoulis M (2003). Nitric oxide and peroxynitrite cause irreversible increases in the K_m_ for oxygen of mitochondrial cytochrome oxidase: in vitro and in vivo studies. *Biochimica et Biophysica Acta*.

[12] Giuffrè A, Sarti P, D’Itri E, Buse G, Soulimane T, Brunori M (1996). On the mechanism of inhibition of cytochrome c oxidase by nitric oxide. *Journal of Biological Chemistry*.

[13] Cooper CE (2002). Nitric oxide and cytochrome oxidase: substrate, inhibitor or effector?. *Trends in Biochemical Sciences*.

[14] Antunes F, Boveris A, Cadenas E (2007). On the biologic role of the reaction of NO with oxidized cytochrome C oxidase. *Antioxidants and Redox Signaling*.

[15] Benard G, Faustin B, Passerieux E (2006). Physiological diversity of mitochondrial oxidative phosphorylation. *American Journal of Physiology*.

[16] Gibson QH, Greenwood C (1963). Reactions of cytochrome oxidase with oxygen and carbon monoxide. *The Biochemical Journal*.

[17] Blackmore RS, Greenwood C, Gibson QH (1991). Studies of the primary oxygen intermediate in the reaction of fully reduced cytochrome oxidase. *Journal of Biological Chemistry*.

[18] Brunori M, Parr SR, Greenwood C, Wilson MT (1975). A temperature jump study of the reaction between azurin and cytochrome c oxidase from *Pseudomonas aeruginosa*. *Biochemical Journal*.

[19] Malatesta F, Sarti P, Antonini G, Vallone B, Brunori M (1990). Electron transfer to the binuclear center in cytochrome oxidase: catalytic significance and evidence for an additional intermediate. *Proceedings of the National Academy of Sciences of the United States of America*.

[20] Verkhovsky MI, Morgan JE, Wikstrom M (1995). Control of electron delivery to the oxygen reduction site of cytochrome c oxidase: a role for protons. *Biochemistry*.

[21] Brunori M, Giuffrè A, D’Itri E, Sarti P (1997). Internal electron transfer in Cu-heme oxidases: thermodynamic or kinetic control. *Journal of Biological Chemistry*.

[22] Sarti P, Forte E, Mastronicola D, Giuffrè A, Arese M (2012). Cytochrome c oxidase and nitric oxide in action: molecular mechanisms and pathophysiological implications. *Biochimica et Biophysica Acta*.

[23] Unitt DC, Hollis VS, Palacios-Callender M, Frakich N, Moncada S (2010). Inactivation of nitric oxide by cytochrome c oxidase under steady-state oxygen conditions. *Biochimica et Biophysica Acta*.

[24] Chance B, Saronio C, Leigh JS (1975). Functional intermediates in the reaction of membrane bound cytochrome oxidase with oxygen. *Journal of Biological Chemistry*.

[25] Weng L, Baker GM (1991). Reaction of hydrogen peroxide with the rapid form of resting cytochrome oxidase. *Biochemistry*.

[26] Fabian M, Wong WW, Gennis RB, Palmer G (1999). Mass spectrometric determination of dioxygen bond splitting in the "peroxy" intermediate of cytochrome c oxidase. *Proceedings of the National Academy of Sciences of the United States of America*.

[27] Han S, Ching YC, Rousseau DL (1990). Ferryl and hydroxy intermediates in the reaction of oxygen with reduced cytochrome c oxidase. *Nature*.

[28] Sarti P, Giuffrè A, Forte E, Mastronicola D, Barone MC, Brunori M (2000). Nitric oxide and cytochrome c oxidase: mechanisms of inhibition and NO degradation. *Biochemical and Biophysical Research Communications*.

[29] Giuffrè A, Barone MC, Mastronicola D, D’Itri E, Sarti P, Brunori M (2000). Reaction of nitric oxide with the turnover intermediates of cytochrome c oxidase: reaction pathway and functional effects. *Biochemistry*.

[30] Stubauer G, Giuffrè A, Brunori M, Sarti P (1998). Cytochrome c oxidase does not catalyze the anaerobic reduction of NO. *Biochemical and Biophysical Research Communications*.

[31] Torres J, Darley-Usmar V, Wilson MT (1995). Inhibition of cytochrome c oxidase in turnover by nitric oxide: mechanism and implications for control of respiration. *Biochemical Journal*.

[32] Mason MG, Nicholls P, Wilson MT, Cooper CE (2006). Nitric oxide inhibition of respiration involves both competitive (heme) and noncompetitive (copper) binding to cytochrome c oxidase. *Proceedings of the National Academy of Sciences of the United States of America*.

[33] Mastronicola D, Arcuri E, Arese M (2004). Morphine but not fentanyl and methadone affects mitochondrial membrane potential by inducing nitric oxide release in glioma cells. *Cellular and Molecular Life Sciences*.

[34] Giuffrè A, Stubauer G, Sarti P (1999). The heme-copper oxidases of *Thermus thermophilus* catalyze the reduction of nitric oxide: evolutionary implications. *Proceedings of the National Academy of Sciences of the United States of America*.

[35] Forte E, Urbani A, Saraste M, Sarti P, Brunori M, Giuffrè A (2001). The cytochrome cbb_3_ from *Pseudomonas stutzeri* displays nitric oxide reductase activity. *European Journal of Biochemistry*.

[36] Butler CS, Forte E, Maria Scandurra F (2002). Cytochrome bo_3_ from *Escherichia coli*: the binding and turnover of nitric oxide. *Biochemical and Biophysical Research Communications*.

[37] Mastronicola D, Genova ML, Arese M (2003). Control of respiration by nitric oxide in Keilin-Hartree particles, mitochondria and SH-SY5Y neuroblastoma cells. *Cellular and Molecular Life Sciences*.

[38] Giuffrè A, Barone MC, Brunori M (2002). Nitric oxide reacts with the single-electron reduced active site of cytochrome c oxidase. *Journal of Biological Chemistry*.

[39] Cooper CE, Mason MG, Nicholls P (2008). A dynamic model of nitric oxide inhibition of mitochondrial cytochrome c oxidase. *Biochimica et Biophysica Acta*.

[40] Brudvig GW, Stevens TH, Chan SI (1980). Reactions of nitric oxide with cytochrome c oxidase. *Biochemistry*.

[41] Cooper CE, Torres J, Sharpe MA, Wilson MT (1997). Nitric oxide ejects electrons from the binuclear centre of cytochrome c oxidase by reacting with oxidised copper: a general mechanism for the interaction of copper proteins with nitric oxide?. *The FEBS Letters*.

[42] Giuffrè A, Stubauer G, Brunori M, Sarti P, Torres J, Wilson MT (1998). Chloride bound to oxidized cytochrome c oxidase controls the reaction with nitric oxide. *Journal of Biological Chemistry*.

[43] Antonini E, Brunori M, Colosimo A (1977). Oxygen ’pulsed’ cytochrome c oxidase: functional properties and catalytic relevance. *Proceedings of the National Academy of Sciences of the United States of America*.

[44] Torres J, Sharpe MA, Rosquist A, Cooper CE, Wilson MT (2000). Cytochrome c oxidase rapidly metabolises nitric oxide to nitrite. *The FEBS Letters*.

[45] Giulivi C (2003). Characterization and function of mitochondrial nitric-oxide synthase. *Free Radical Biology and Medicine*.

[46] Pearce LL, Kanai AJ, Birder LA, Pitt BR, Peterson J (2002). The catabolic fate of nitric oxide. The nitric oxide oxidase and peroxynitrite reductase activities of cytochrome oxidase. *Journal of Biological Chemistry*.

[47] Sgarbi G, Baracca A, Lenaz G, Valentino LM, Carelli V, Solaini G (2006). Inefficient coupling between proton transport and ATP synthesis may be the pathogenic mechanism for NARP and Leigh syndrome resulting from the T8993G mutation in mtDNA. *Biochemical Journal*.

[48] Arese M, Magnifico MC, Mastronicola D (2012). Nanomolar melatonin enhances nNOS expression and controls HaCaT-cells bioenergetics. *IUBMB Life*.

[49] Merlo-Pich M, Deleonardi G, Biondi A, Lenaz G (2004). Methods to detect mitochondrial function. *Experimental Gerontology*.

[50] Giuffrè A, Forte E, Brunori M, Sarti P (2005). Nitric oxide, cytochrome c oxidase and myoglobin: competition and reaction pathways. *The FEBS Letters*.

[51] Borisov VB, Forte E, Giuffrè A, Konstantinov A, Sarti P (2009). Reaction of nitric oxide with the oxidized di-heme and heme-copper oxygen-reducing centers of terminal oxidases: different reaction pathways and end-products. *Journal of Inorganic Biochemistry*.

[52] Borisov VB, Forte E, Konstantinov AA, Poole RK, Sarti P, Giuffrè A (2004). Interaction of the bacterial terminal oxidase cytochrome bd with nitric oxide. *The FEBS Letters*.

[53] Borisov VB, Forte E, Sarti P, Brunori M, Konstantinov AA, Giuffrè A (2007). Redox control of fast ligand dissociation from *Escherichia coli* cytochrome bd. *Biochemical and Biophysical Research Communications*.

[54] Rinaldo S, Sam KA, Castiglione N (2011). Observation of fast release of NO from ferrous d1 haem allows formulation of a unified reaction mechanism for cytochrome cd1 nitrite reductases. *Biochemical Journal*.

[55] Mason MG, Nicholls P, Cooper CE (2009). The steady-state mechanism of cytochrome c oxidase: redox interactions between metal centres. *Biochemical Journal*.

[56] Giuffrè A, Borisov VB, Mastronicola D, Sarti P, Forte E (2012). Cytochrome bd oxidase and nitric oxide: from reaction mechanisms to bacterial physiology. *The FEBS Letters*.

[57] Masci A, Mastronicola D, Arese M (2008). Control of cell respiration by nitric oxide in Ataxia Telangiectasia lymphoblastoid cells. *Biochimica et Biophysica Acta*.

[58] Torres J, Cooper CE, Wilson MT (1998). A common mechanism for the interaction of nitric oxide with the oxidized binuclear centre and oxygen intermediates of cytochrome c oxidase. *Journal of Biological Chemistry*.

[59] Lundberg JO, Weitzberg E, Gladwin MT (2008). The nitrate-nitrite-nitric oxide pathway in physiology and therapeutics. *Nature Reviews Drug Discovery*.

[60] Shiva S, Gladwin MT (2009). Nitrite mediates cytoprotection after ischemia/reperfusion by modulating mitochondrial function. *Basic Research in Cardiology*.

[61] Webb A, Bond R, McLean P, Uppal R, Benjamin N, Ahluwalia A (2004). Reduction of nitrite to nitric oxide during ischemia protects against myocardial ischemia-reperfusion damage. *Proceedings of the National Academy of Sciences of the United States of America*.

[62] Zweier JL, Samouilov A, Kuppusamy P (1999). Non-enzymatic nitric oxide synthesis in biological systems. *Biochimica et Biophysica Acta*.

[63] Whorton AR, Simonds DB, Piantadosi CA (1997). Regulation of nitric oxide synthesis by oxygen in vascular endothelial cells. *American Journal of Physiology*.

[64] Poyton RO, Castello PR, Ball KA, Woo DK, Pan N (2009). Mitochondria and hypoxic signaling: a new view. *Annals of the New York Academy of Sciences*.

[65] Stubauer G, Giuffrè A, Sarti P (1999). Mechanism of S-nitrosothiol formation and degradation mediated by copper ions. *Journal of Biological Chemistry*.

[66] Dedkova EN, Ji X, Lipsius SL, Blatter LA (2004). Mitochondrial calcium uptake stimulates nitric oxide production in mitochondria of bovine vascular endothelial cells. *American Journal of Physiology*.

[67] Brown GC (1995). Nitric oxide produced by activated astrocytes rapidly and reversibly inhibits cellular respiration. *Neuroscience Letters*.

[68] Mercadante S (2011). Managing breakthrough pain. *Current Pain and Headache Reports*.

[69] Plante GE, Vanitallie TB (2010). Opioids for cancer pain: the challenge of optimizing treatment. *Metabolism*.

[70] Pasternak GW, Kolesnikov YA, Babey AM (1995). Perspectives on the N-methyl-D-aspartate/nitric oxide cascade and opioid tolerance. *Neuropsychopharmacology*.

[71] Mark Quillan J, Carlson KW, Song C, Wang D, Sadée W (2002). Differential effects of *μ*-opioid receptor ligands on Ca^2+^ signaling. *Journal of Pharmacology and Experimental Therapeutics*.

[72] Reers M, Smith TW, Chen LB (1991). J-aggregate formation of a carbocyanine as a quantitative fluorescent indicator of membrane potential. *Biochemistry*.

[73] Luchetti F, Canonico B, Betti M (2010). Melatonin signaling and cell protection function. *The FASEB Journal*.

[74] Warburg O (1956). On respiratory impairment in cancer cells. *Science*.

[75] Brookes PS, Kraus DW, Shiva S (2003). Control of mitochondrial respiration by NO^*·*^, Effects of low oxygen and respiratory state. *Journal of Biological Chemistry*.

[76] Borutaité V, Brown GC (1996). Rapid reduction of nitric oxide by mitochondria, and reversible inhibition of mitochondrial respiration by nitric oxide. *Biochemical Journal*.

[77] Palacios-Callender M, Hollis V, Frakich N, Mateo J, Moncada S (2007). Cytochrome c oxidase maintains mitochondrial respiration during partial inhibition by nitric oxide. *Journal of Cell Science*.

[78] Palacios-Callender M, Hollis V, Mitchison M, Frakich N, Unitt D, Moncada S (2007). Cytochrome c oxidase regulates endogenous nitric oxide availability in respiring cells: a possible explanation for hypoxic vasodilation. *Proceedings of the National Academy of Sciences of the United States of America*.

[79] Chance B (1965). Reaction of oxygen with the respiratory chain in cells and tissues. *Journal of General Physiology*.

[80] Mazat JP, Rossignol R, Malgat M, Rocher C, Faustin B, Letellier T (2001). What do mitochondrial diseases teach us about normal mitochondrial functions... that we already knew: threshold expression of mitochondrial defects. *Biochimica et Biophysica Acta*.

[81] Brookes PS, Bolaños JP, Heales SJR (1999). The assumption that nitric oxide inhibits mitochondrial ATP synthesis is correct. *The FEBS Letters*.

[82] Bolaños JP, Almeida A, Moncada S (2010). Glycolysis: a bioenergetic or a survival pathway?. *Trends in Biochemical Sciences*.

[83] Almeida A, Almeida J, Bolaños JP, Moncada S (2001). Different responses of astrocytes and neurons to nitric oxide: the role of glycolytically generated ATP in astrocyte protection. *Proceedings of the National Academy of Sciences of the United States of America*.

[84] Antunes F, Boveris A, Cadenas E (2004). On the mechanism and biology of cytochrome oxidase inhibition by nitric oxide. *Proceedings of the National Academy of Sciences of the United States of America*.

[85] Meyn MS (1999). Ataxia-telangiectasia, cancer and the pathobiology of the ATM gene. *Clinical Genetics*.

[86] McKinnon PJ (2004). ATM and ataxia telangiectasia. *EMBO Reports*.

[87] Savitsky K, Sfez S, Tagle DA (1995). The complete sequence of the coding region of the ATM gene reveals similarity to cell cycle regulators in different species. *Human Molecular Genetics*.

[88] Rotman G, Shiloh Y (1998). ATM: from gene to function. *Human Molecular Genetics*.

[89] Lavin MF, Shiloh Y (1997). The genetic defect in ataxia-telangiectasia. *Annual Review of Immunology*.

[90] Barzilai A, Rotman G, Shiloh Y (2002). ATM deficiency and oxidative stress: a new dimension of defective response to DNA damage. *DNA Repair*.

[91] Reliene R, Fischer E, Schiestl RH (2004). Effect of N-acetyl cysteine on oxidative DNA damage and the frequency of DNA deletions in Atm-deficient mice. *Cancer Research*.

[92] Barlow C, Hirotsune S, Paylor R (1996). Atm-deficient mice: a paradigm of ataxia telangiectasia. *Cell*.

[93] Xu Y, Ashley T, Brainerd EE, Bronson RT, Meyn MS, Baltimore D (1996). Targeted disruption of ATM leads to growth retardation, chromosomal fragmentation during meiosis, immune defects, and thymic lymphoma. *Genes and Development*.

[94] Bishop AJR, Barlow C, Wynshaw-Boris AJ, Schiestl RH (2000). Atm deficiency causes an increased frequency of intrachromosomal homologous recombination in mice. *Cancer Research*.

[95] Reichenbach J, Schubert R, Schindler D, Müller K, Böhles H, Zielen S (2002). Elevated oxidative stress in patients with Ataxia telangiectasia. *Antioxidants and Redox Signaling*.

[96] Aksoy Y, Sanal O, Metin A (2004). Antioxidant enzymes in red blood cells and lymphocytes of ataxia-telangiectasia patients. *Turkish Journal of Pediatrics*.

[97] Ambrose M, Goldstine JV, Gatti RA (2007). Intrinsic mitochondrial dysfunction in ATM-deficient lymphoblastoid cells. *Human Molecular Genetics*.

